# Supplementary material for: A Comprehensive Assessment of Ultraviolet-Radiation-Induced Mutations in Flammulina filiformis Using Whole-Genome Resequencing
Source: J Fungi (Basel). 2024 Mar 20;10(3):228. doi: 10.3390/jof10030228 (PMC10971301; doi:10.3390/jof10030228)
Supplement: Supplementary file 1 [file jof-10-00228-s001.zip › Supplementary Material S8/KEGG annotation/out/64381550635650.os/KO/out_map/map04611.html]

KEGG PATHWAY: Platelet activation - Reference pathway


|  |  |
| --- | --- |
| **Platelet activation - Reference pathway** |  |

[
Pathway menu
| Organism menu
| Pathway entry
| Show description
| User data mapping
]

Platelets play a key and beneficial role for primary hemostasis on the disruption of the integrity of vessel wall. Platelet adhesion and activation at sites of vascular wall injury is initiated by adhesion to adhesive macromolecules, such as collagen and von Willebrand factor (vWF), or by soluble platelet agonists, such as ADP, thrombin, and thromboxane A2. Different receptors are stimulated by various agonists, almost converging in increasing intracellular Ca2+ concentration that stimulate platelet shape change and granule secretion and ultimately induce the "inside-out" signaling process leading to activation of the ligand-binding function of integrin alpha IIb beta 3. Binding of alpha IIb beta 3 to its ligands, mainly fibrinogen, mediates platelet adhesion and aggregation and triggers "outside-in" signaling, resulting in platelet spreading, additional granule secretion, stabilization of platelet adhesion and aggregation, and clot retraction.

|  |  |
| --- | --- |
| Reference pathway | 100% |
